# Radiomic Features Are Predictive of Response in Rectal Cancer Undergoing Therapy

**DOI:** 10.3390/diagnostics13152573

**Published:** 2023-08-02

**Authors:** Diletta Santini, Ginevra Danti, Eleonora Bicci, Antonio Galluzzo, Silvia Bettarini, Simone Busoni, Tommaso Innocenti, Andrea Galli, Vittorio Miele

**Affiliations:** 1 Department of Radiology, Careggi University Hospital, Largo Brambilla 3, 50134 Florence, Italy; diletta.santini08@gmail.com (D.S.);; 2 Department of Health Physics, Careggi University Hospital, Largo Brambilla 3, 50134 Florence, Italy; 3 Clinical Gastroenterology Unit, Careggi University Hospital, Largo Brambilla 3, 50134 Florence, Italy

**Keywords:** rectal cancer, locally advanced rectal cancer, pathological complete response, chemoradiotherapy, magnetic resonance imaging, radiomic

## Abstract

Background: Rectal cancer is a major mortality cause in the United States (US), and its treatment is based on individual risk factors for recurrence in each patient. In patients with rectal cancer, accurate assessment of response to chemoradiotherapy has increased in importance as the variety of treatment options has grown. In this scenario, a controversial non-operative approach may be considered in some patients for whom complete tumor regression is believed to have occurred. The recommended treatment for locally advanced rectal cancer (LARC, T3-4 ± N+) is total mesorectal excision (TME) after neoadjuvant chemoradiotherapy (nCRT). Magnetic resonance imaging (MRI) has become a standard technique for local staging of rectal cancer (tumor, lymph node, and circumferential resection margin [CRM] staging), in both the US and Europe, and it is getting widely used for restaging purposes. Aim: In our study, we aimed to use an MRI radiomic model to identify features linked to the different responses of chemoradiotherapy of rectal cancer before surgery, and whether these features are helpful to understand the effectiveness of the treatments. Methods: We retrospectively evaluated adult patients diagnosed with LARC who were subjected to at least 2 MRI examinations in 10–12 weeks at our hospital, before and after nCRT. The MRI acquisition protocol for the 2 exams included T2 sequence and apparent diffusion coefficient (ADC) map. The patients were divided into 2 groups according to the treatment response: complete or good responders (Group 1) and incomplete or poor responders (Group 2). MRI images were segmented, and quantitative features were extracted and compared between the two groups. Features that showed significant differences (SF) were then included in a LASSO regression method to build a radiomic-based predictive model. Results: We included 38 patients (26 males and 12 females), who are classified from T2 and T4 stages in the rectal cancer TNM. After the nCRT, the patients were divided into Group 1 (13 patients), complete or good responders, and Group 2 (25 patients), incomplete or poor responders. Analysis at baseline generated the following significant features for the Mann–Whitney test (out of a total of 107) for each sequence. Also, the analysis at the end of the follow-up yielded a high number of significant features for the Mann–Whitney test (out of a total of 107) for each image. Features selected by the LASSO regression method for each image analyzed; ROC curves relative to each model are represented. Conclusion: We developed an MRI-based radiomic model that is able to differentiate and predict between responders and non-responders who went through nCRT for rectal cancer. This approach might identify early lesions with high surgical potential from lesions potentially resolving after medical treatment.

## 1. Introduction

Colorectal cancer is a major cause of mortality in the United States [1], it is considered the third top malignancy in the world [2]; rectal cancer covers approximately 15–35% of whole colorectal cancer [3]. The more developed countries show the highest incidence of cases compared to the less developed countries. Treatment of patients with rectal cancer is based on individual risk factors for recurrence in each patient. The rectal cancer is staged by TNM, and individuals staging from T3 to T4, with or without positive lymph node, are normally recommended with neoadjuvant treatment. The recommended treatment for locally advanced rectal cancer (LARC, T3-4 ± N+) is a total mesorectal excision (TME) after neoadjuvant chemoradiotherapy (nCRT) [4]. However, LARC shows variable response rates to nCRT for different patients, ranging from no tumor regression to pathologic complete response (pCR) [5]. Habr-Gama et al. [6] showed that selected patients with a clinical complete response to CRT can be safely followed up in a nonsurgical approach. Accurate restaging is crucial for patients with LARC undergoing neoadjuvant treatment because the identification of response has major implications for management [7]. 

Magnetic resonance imaging (MRI) is a standard technique for local staging of rectal cancer (tumor, lymph node, and circumferential resection margin [CRM] staging) and is also increasingly used for restaging [8]. In primary staging (pre-therapeutic setting), MRI can assist with the selection of patients with LARC who are suitable for treatment with nCRT; guiding surgeons in surgical planning; and identifying poor prognostic factors, including extramural vascular invasion (EMVI), mucin content, and involvement of the mesorectal fascia (MRF) [9]. In the restaging setting (after treatment with nCRT), rectal MRI can help to evaluate the tumor regression, tailoring surgical planning and detecting a complete clinical response, along with a review of the results of digital rectal examinations and endoscopic procedures and monitoring patients undergoing the non-surgical treatment approach. Rectal cancer staging is based on the TNM staging system and the criteria of the American Joint Committee on Cancer (AJCC) [10]. MRI has been shown to be an accurate means to evaluate the involvement of the circumferential resection margin, with a sensitivity of 87% and specificity of 75% for T1 and T2 tumors, and with a sensitivity of 77% and specificity of 94% for T3 and T4 tumors [11]. 

The primary role of CT in rectal cancer is for M staging, with common sites of metastatic spread being the liver, lungs, nervous system, bone, and peritoneal surfaces [12]. Surgery plays a pivotal role in the initial management of rectal cancer and may qualify as the sole treatment in certain cases, especially for early-stage cancers. For more advanced cancers, TME is pursued when surgical management is warranted, either without or after nCRT [13,14]. The “watch-and-wait” approach to treatment after chemoradiation therapy is a potential option for a select minority of patients. This strategy consists of the close observation of the patients who have a clinical complete response to nCRT and wait before moving straight to surgery. The goal is to avoid surgical morbidity. Reported surveillance includes close evaluation with physical examination, endoscopy, and MRI every 3 months. [15]. Analysis of morphologic changes in tumor seems to be essential for the evaluation of tumors after the chemoradiation therapy and to assess treatment response. Several morphologic responses at histopathologic examination have been described after chemoradiation therapy, including fibrosis replacing tumor glands with or without prominent inflammatory cells, a lack of tumor necrosis, and increased mucin production, all of which demonstrate distinct findings at imaging [16]. 

Radiomics, which is based on advanced pattern recognition tools, involves the extraction of a large number of quantitative features from digital images to determine the relationships between such features and the underlying pathophysiology [17]. Radiomics has been shown to carry the potential of both predicting and evaluating responses to CRT [18]. In our study, we investigate whether there are some quantitative MRI-based radiomics features able to predict the response to nCRT, in order to stratify patients into potential good and poor responders to nCRT at the staging time. 

## 2. Methods

### 2.1. Study Population

The study was a retrospective analysis of MRI images of patients who were referred to our Hospital (Radiology Department of Careggi University Hospital, Florence, Italy) for a rectal cancer follow-up from March 2020 to March 2023. The adult patients with known rectal cancer who had had at least two MRI studies were followed up at our hospital in 10–12 weeks, according to Santiago et al. [19], before and after radiochemotherapy. 

Neoadjuvant therapy comprises radiotherapy in combination with chemotherapy. Commonly prescribed chemotherapy agents include 5-Fluorouracil (5-FU) or Capecitabine. When it comes to advanced cancer, it is helpful to start with strong therapy that comprises folic acid, fluorouracil, and oxaliplatin (FOLFOX). All our patients underwent long-course radiotherapy (45–50 Gy in 25–28 fractions, administered 5 days per week for the duration of radiation) concurrently with chemotherapy. After the follow-up MRI, surgical treatment was performed as needed. The radiation clinical target volume (CTV) included the primary rectal cancer, perirectal and internal iliac nodes, mesorectum, pelvic sidewalls, and presacral space with the upper border at the sacral promontory. 

Inclusion criteria were: (1) diagnosis of rectal cancer (2) and having an MRI evaluation pre- and post-radiochemotherapy archived in our picture archiving and communication systems (PACS). Exclusion criteria were: (1) pediatric age (<18 years), (2) unavailability of both MRI exams pre- and post-radiochemotherapy, (3) and unavailability of one or more of the selected sequences including T2-weighted (T2-W) and apparent diffusion coefficient (ADC) map. At the time of the follow-up pre-surgery, the patients were divided into two groups based on the different responses of the neoadjuvant therapy: complete or good responders (Group 1) and incomplete or poor responders (Group 2). For each patient, we considered age, sex, TNM rectal cancer staging, CRM, and EMVI.

### 2.2. Pathological Case Selection and Classification

Patients who qualify as locally advanced rectal cancer (LARC, T3-4 ± N+) are recommended a neoadjuvant therapy before the possibility of TME [4]. In particular, the T3 tumor extends through the muscularis propria and into the mesorectal tissues. T4 tumor denotes invasion into adjacent organs, and the invasion could be either confined to only the peritoneal reflection (T4a) or expanded to other organs or structures (T4b). Nodal (N) staging is determined by the presence and number of the regional lymph nodes involved, which are the mesorectal and internal iliac nodal stations [13]. This study includes only patients until stage T4a N+ and they could present cancer either in the inferior or middle or upper rectum. None of them have the anal sphincter involved with tumor. Clinical features such as age and sex were reported by clinical reports.

### 2.3. Imaging Acquisition 

**Figure 1:**
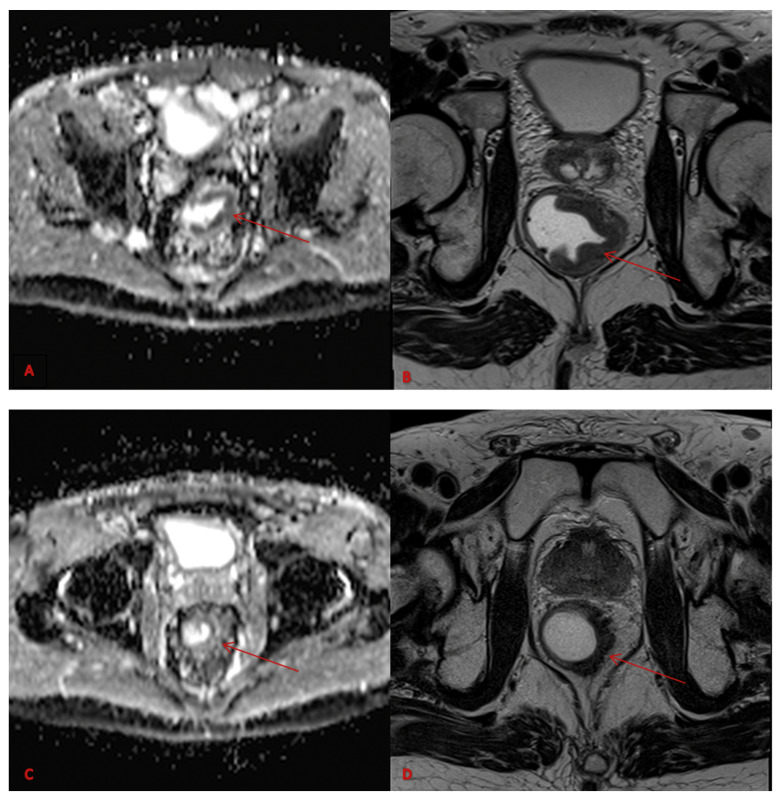
ADC map pre-therapy (**A**), Axial MRI T2w pre-therapy (**B**), ADC map post-therapy (**C**) and Axial MRI T2w post-therapy (**D**). Images refer to rectal cancer (red arrows).

MRI examinations were performed on a Siemens [MR Systems Aera and Magnetom Sola, SRN:84081] 1.5 T scanner and external coils for the inferior abdomen. The MRI acquisition protocol for both baseline and follow-up included the subsequent sequences: high-resolution T2-WI a slice thickness ≤ 3 mm (FSE 2D without fat saturation; TR:3800 ms, TE: 101 ms) in oblique axial sequence to the tumor, sagittal and oblique coronal planes to the rectal canal (small FOV 180 mm), oblique axial DWI (DWI_3b_0-500-1000, small FOV:320 mm) with ADC map reconstruction (EPI; TR:6800 ms, TE:56 ms), and T1 pelvic axial sequence (large FOV:310 mm, TR:453 ms, TE:7.8 ms) to examine the iliac and paraortic lymph nodes. Intravenous paramagnetic contrast administration is not routinely recommended [20]. Each patient is asked to perform a small enema to empty the rectum the day prior to the examination [21], and 50 mL of endoluminal gel is given to prevent excessive compression of the mesorectal fat during the examination (Figure 1). These two steps are critical for the final quality of the imaging. 

### 2.4. Imaging Analysis 

Two radiologists, with at least three years of experience in abdominal radiology, independently reviewed the MRI images blinded to clinical-laboratory data under the supervision of a radiologist with more than ten years of experience, who reviewed the images in the event of non-concordance. Image analysis was conducted in two steps: first, pre- and post-neoadjuvant therapy exams were compared for each patient. Then, the patients were divided into 2 groups based on their response to therapy: complete or good responders (Group 1) and incomplete or not responders (Group 2). For each patient, 2 MRI examinations at least 10–12 weeks apart were retrieved from our PACS.

### 2.5. Radiomic Workflow

**Figure 2:**
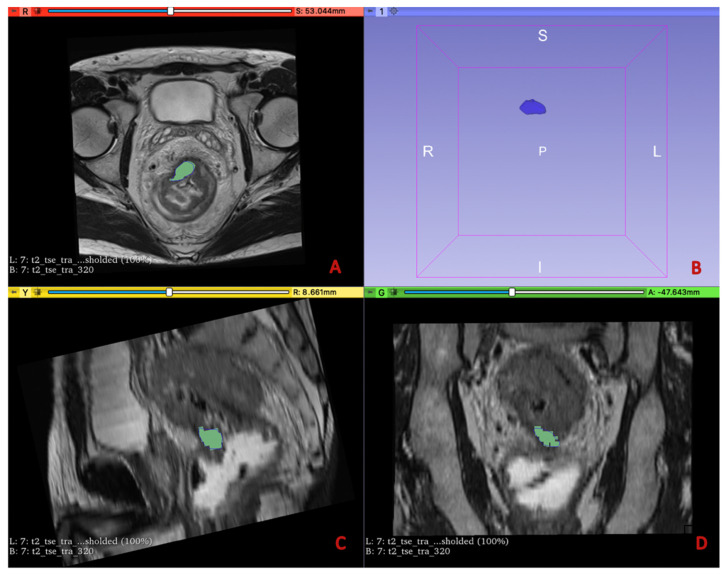
The image-processing software 3D Slicer was used to manually outline ROIs along the lesion margins on all the slices containing the tumor in the MRI T2w sequences (**A**,**C**,**D**) and ADC sequences, as shown in the figure. The volumetric 3D tumor reconstruction is shown in the upper right quadrant (**B**).

The radiomic workflow included: [1] lesion segmentation (Figure 2), feature extraction [2,3] feature selection. Subsequently, pre- and post-neoadjuvant therapy T2-W and ADC map images were loaded into 3D Slicer software, version 4.10.2 (open-source software; https://www.slicer.org/ accessed on 21 June 2023) for segmentation. The radiologists manually delineated the tumor area on all slices for each sequence by defining the region of interest (ROI). With SlicerRadiomics extension, implemented in 3D Slicer software, a total of 107 quantitative radiomic features were extracted from each ROI. The extracted features were subdivided into 3 main classes: “shape-based” (in our case only the 3D type), first-order statistics, and second-order statistics. Briefly, “shape-based” features are descriptors of the three-dimensional size and shape of the ROI, “first-order (I-order) statistics” features represent the distribution of voxel intensities within the ROI, and “second-order (II-order) statistics” features provide the spatial arrangement correlation of the intensity values within the ROI. Through statistical analysis, the significant features were then selected and the model was built for each sequence in pre- and post-nCRT MR examinations.

### 2.6. Statistical Analysis

Non-parametric Mann–Whitney test was performed in the R software, version 4.1.1 (open-source software, https://www.r-project.org/ accessed on 21 June 2023), to identify features that showed statistically significant differences between the 2 groups. A *p*-value < 0.05 was considered significant. The radiomic-based model was built in R using the least absolute shrinkage and selection operator (LASSO) regression method, which allows the same time to make feature reduction and selection. Along with the radiomic model, Receiver Operating Characteristic (ROC) curves and Area Under the Curve (AUC) values were generated and a 95% confidence interval (CI) was reported. This statistical analysis was carried out for T2-w sequences and ADC maps for both pre- and post-neoadjuvant therapy MR examinations.

### 2.7. Compliance with Ethical Standards

The authors declare no conflict of interest. All patients signed an informed consent form. The study was approved by the Biomedical Research Ethics Committee of our institution in accordance with the criteria of the Declaration of Helsinki on Ethical Principles and Good Clinical Practice.

## 3. Results

A totally of 70 patients with known rectal cancer, who underwent MRI between March 2020 and March 2023, with a median follow-up time of 10–12 weeks, were selected. Among these, thirty-two patients did not have good-quality radiological images. They either did not have examinations from the same MRI scanner or were tested for MRI follow-ups by two different machines. As a result of this, they were excluded from the analysis. A final number of 38 patients was included in our study. Baseline and demographic characteristics of patients are reported in Table 1. In our study, we enrolled 26 males and 12 females, and the average age at the diagnosis was 66, 9 years old. According to TNM staging, 2 (5.2%) out of our patients were classified as stage T2 at the baseline exam, 27 (71%) were classified as stage T3, and 9 (23.8%) as stage T4. Among the patients, we also analyzed that 20 (52.6%) of them were CRM positive and 9 (23.8%) of them were EMVI positive. Mucinous tumors, at the beginning of the diagnosis, were 6 (15.8%). After the nCRT, the patients were divided into Group 1, complete or good responders, (13 patients) and Group 2, incomplete or poor responders, (25 patients). Out of group 1, made of 13 patients, only 1 patient responded to a mucinous answer. We want to remember that such type of answer is considered a complete response to treatment.

In this first group, there were 2 stage T2 patients (based on TNM Staging) at the baseline exam, 10 patients who were classified as stage T3, and only 1 patient was diagnosed as stage T4. In the group 2, made of 25 patients, there were 6 patients with mucinous tumor. As in most of the literature, they were included in the group of bad or non-responders. 

In this second group, none were classified as stage T2 at the baseline exam, 18 patients were classified as stage T3, and 7 patients were classified as stage T4 (Table 2). 

Analysis at baseline (pre-nCRT) generated the following significant features for the Mann–Whitney test (out of a total of 107) for each sequence: 26 features for the ADC map, of these 1 of the shape features present, and 25 II-order features; 16 features for the T2-W sequence, all II-order features. Analysis at the end of follow-up (post-nCRT) yielded a high number of significant features for the Mann–Whitney test (out of a total of 107) for each image (Supplementary Table S1): 32 features for the ADC map, of which 8 shape features and 24 II-order features; 7 features for the T2-W sequence, of which 2 shape features and 5 of the II-order features. 

**Figure 3:**
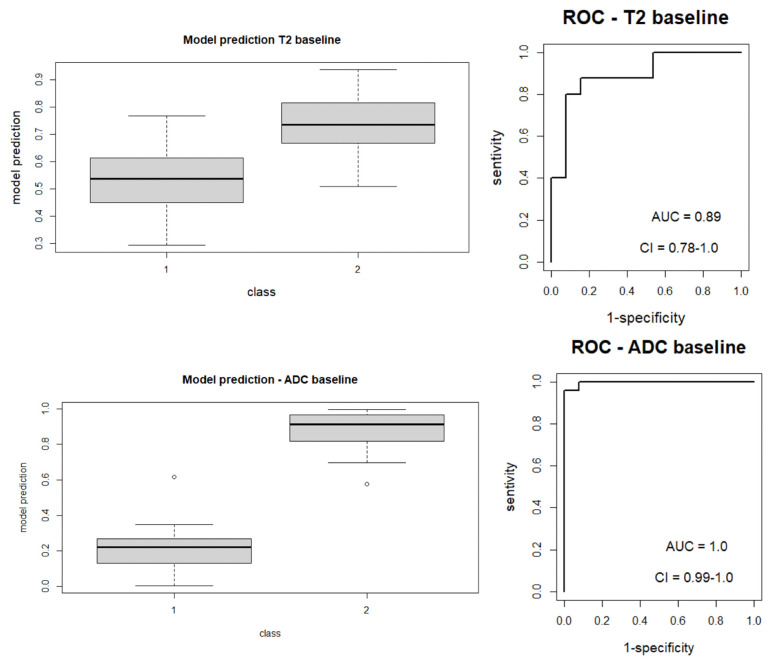
Boxplot of performance in prediction models (**left**) and ROC curves (with AUC) associated with prediction models (**right**); prediction models were calculated with the LASSO regression method on baseline images (T2 and ADC map).

**Figure 4:**
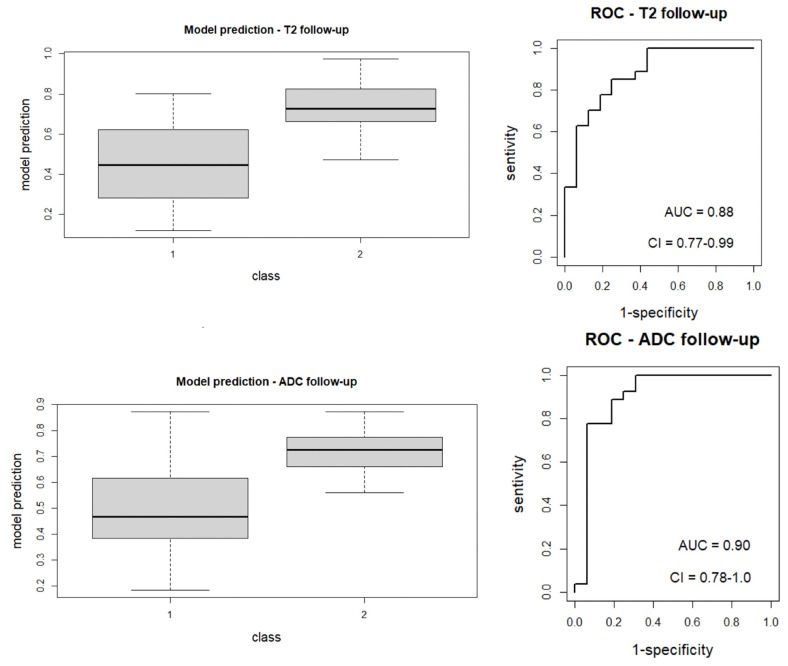
Boxplot of performance in prediction models (**left**) and ROC curves (with AUC) associated with models (**right**); prediction models were calculated with the LASSO regression method on the follow-up images (T2 and ADC map) after nCRT.

Features selected by the LASSO regression method are reported in Table 3 for each MRI image analyzed, which differentiates between responders and non-responders to nCRT; ROC curves relative to each model are represented in Figure 3 and Figure 4. Figure 3 represents a boxplot of performance in prediction models (left) and ROC curves (with AUC) associated with prediction models (right); prediction models were calculated with the LASSO regression method on baseline images (T2 and ADC map). Figure 4 portrays a boxplot of performance in prediction models (left) and ROC curves (with AUC) associated with models (right); prediction models were calculated with the LASSO regression method on the follow-up images (T2 and ADC map) after nCRT. Analyzing the MRI images at the baseline, the following features were selected for each radiomic model (reported with AUC value). For the ADC map Sphericity, Elongation, Surface Volume Ratio, Flatness (shape features), Cluster Shade, Joint Energy, Difference Variance, ID, Minimum, Kurtosis, and Size Zone Non-Uniformity (second order features) with AUC value = 1.0 (95%CI: 0.99–1. 00); for the T2-W sequence Sphericity (shape feature), Dependence Non-Uniformity Normalized, Large Dependence Emphasis, Size Zone Non-Uniformity Normalized, and Busyness (second-order features) with AUC value = 0.89 (95%CI: 0.78–1.00). Repeating the analysis on the MRI sequences after treatment, the features selected by the radiomic model and the AUC associated with the ROC, for each sequence, were the following: for the ADC map Sphericity and Elongation (shape features), Busyness, and Low Gray Level Run Emphasis (second-order features) with AUC value = 0. 90 (95%CI: 0.78–1.00); for the T2-W sequence Flatness (shape features), Long Run High Gray Level Emphasis, Size Zone Non-Uniformity Normalized, and Strength (second-order features) with AUC value = 0.88 (95%CI: 0.77–0.99). 

## 4. Discussion

In this study, we developed an MRI-based radiomic model that is able to differentiate and predict between responders and non-responders rectal cancer patients undergoing nCRT. The distinction between these two groups is extremely important to determine further treatment plans. It may be an effective tool to evaluate radiochemotherapy outcomes in patients with LARC. After nCRT, approximately 50% to 60% of patients are downstaged and 15% to 27% show pCR; although the assessment of clinical complete response (cCR) after nCRT has become increasingly important as well, especially in non-surgical management where accurate diagnosis is essential [21,22].

As we mentioned before, several histopathological features of response, including fibrosis, a lack of tumor necrosis, mucin production, or residual tumor [16]. According to the most recent literature [19,23,24,25,26], the rectal cancers, diagnosed as mucinous, show an incomplete or non-response to the neoadjuvant treatment. For these reasons, all patients with mucinous neoplasms were included in Group 2. Only one of our patients showed mucinous changes after chemoradiation therapy as a sign of treatment response [13,22,23]. 

Radiomics could be a helpful method to stratify responders and non-responders after treatment. A large prospective trial in the MRI and Rectal Cancer European Equivalence (MERCURY) study showed that standard morphological MRI (*T2*) is associated with survival outcomes [27], indicating the important role of post-nCRT MRI assessment of tumor regression grade in prognosis. There are some radiomics studies about post-nCRT evaluation, most of them based on T2-W sequences and ADC map. Yuqiang Li et al. obtained MRI-based radiomic features from axial T2 weighted images in 165 patients, their study showed that a radiomic predictive model was promising to predict pCR to nCRT in LARC patients. The evaluation of T2 sequences, along with clinical information, seems to be a pragmatic approach to strategy-making [27]. In the study by Aytul Hande Yardimci et al., 76 patients with clinicopathologically confirmed LARC, who received nCRT were enrolled, and radiomic model was developed. More particularly, the authors developed machine-learning-based clinical, radiomic, and combined models for predicting therapeutic response to nCRT in patients with LARC; their analysis focused on the basal sagittal plane using a 3D volumetric technique, in contrast with many previous studies which used axial plane images. They also used clinical and laboratory data to build predictive models [28]. Other research by Yuan Cheng et al. built an MRI-based radiomic nomogram able to predict response to nCRT in 193 patients with confirmed LARC who received nCRT treatment before surgery. All their patients underwent baseline T1-weighted (T1W), T2-weighted (T2W), and T2-weighted fat- suppression (T2FS) MRI scans before nCRT [27,29].

In our study, we developed a radiomic model for noninvasive, locally advanced, rectal cancer based on pre- and post-neoadjuvant treatment MRI data. We identified some radiomic features which can predict the two main different answers to the treatment. We compared the patients with complete or good responses (Group 1) to patients with incomplete or non-response (Group 2); a high number of significant features in the distinction between the 2 groups emerged from the analysis on the 2 MRI sequences analyzed, namely T2 and ADC map. The same analysis was repeated on both baseline and follow-up MRI exams, obtaining many significant features. From both MRI exams, baseline and follow-up, we obtained shape and II-order features. In particular, the resulting significant features in the distinction between the two groups on baseline images, thus already present before the treatment, could help us to predict treatment response. Through the LASSO regression method, in fact, it was then possible to build a predictive model with a good AUC value, even on the images from the baseline. The features selected by the radiomic model on baseline ADC map images as follows: Sphericity, Elongation, Surface Volume Ratio and Flatness (shape features), Cluster Shade, Joint Energy, Difference Variance and Size Zone Non-Uniformity (II order features), and Minimum and Kurtosis (I order features). For the T2 w baseline sequences model, the selected features are: Flatness and Strength (shape features), Long Run High Gray Level Emphasis, and Size Zone Non-Uniformity Normalized (II order features). These features are able to identify the good and poor responders to the therapy. These results could facilitate the selection of patients requiring surgery or those who only need a “watch and wait approach”. Our results are comparable to those of other studies that were taken into consideration, and the final goal is to predict patient management before starting the treatments. Using the radiomic model built on baseline images, it could be possible to identify the patients who need surgery, after the neoadjuvant treatment, through a non-invasive and quantitative method. 

Our study has many inherent limitations, relating both to the relatively small number of patients evaluated and the monocentric and retrospective analysis aspects. Also, we did not perform an external validation test to demonstrate the reproducibility of the results. On the other hand, our small sample size is due to our decision to only include patients with high-quality imaging data, in order to make the radiomic model more robust, which is the greater strength of our work. In the future, it will be interesting to expand the number of patients examined to test the accuracy of the data collected from a larger cohort, correlate them with different nCRT regimens, and include clinical data to have the opportunity to develop a nomogram. 

In conclusion, our MRI-based radiomic model can predict treatment response in LARC patients. This approach might early identify lesions with high surgical potential from lesions that may be resolved after medical treatment. This could be applied in clinical practice, reducing the need for surgery for some patients, or predicting the patient management at the time of diagnosis. In the future, we hope to find an easy and practical model to be used in clinical practice. 

## Figures and Tables

**Table 1:** Demographic characteristics of the population at the baseline.

	N = 38
Males	26
Females	12
Median age at diagnosis (IQR)	66.9
T2 *	2
T3 *	27
T4 *	9
CRM positive	20
EMVI positive	9
Mucinous tumor	6
Median follow-up [weeks (IQR)]	

* According to TNM criteria.

**Table 2:** Group 1 and 2 after neoadjuvant radiochemotherapy.

	N = 38
Good responders (group 1)	13
- Mucinous response	1
- Mucinous tumor	0
- T2 *	2
- T3 *	10
- T4 *	1
Poor responders (group 2)	25
- Mucinous tumor	6
- T2 *	0
- T3 *	18
- T4 *	7

* According to TNM criteria.

**Table 3:** Radiomic features selected by the LASSO regression method for each MRI phase that differentiate between responders and non-responders to nCRT.

MRI Phases	Selected Features	Level/Order
T2 baseline	Dependence Non Uniformity Normalized	Second-order
	Size Zone Non Uniformity Normalized	Second-order
	Busyness	Second-Order
	Sphericity	Shape
	Large Dependence Emphasis	Second-order
T2 follow-up	Flatness	Shape
	Strenght	Shape
	Size Zone Non Uniformity Normalized	Second-order
	LongRunHighGrayLevelEmphasis	Second-order
ADC baseline	Difference Variance	Second-order
	Id	Second-order
	Sphericity	Shape
	Elongation	Shape
	Surface volume Ratio	Shape
	Flatness	Shape
	Cluster Shade	Second-order
	Joint Energy	Second-order
	Minimun	First-order
	Kurtosis	First-order
	Size Zone Non Uniformity Normalized	Second-order
ADC follow up	Sphericity	Shape
	Elongation	Shape
	Low Gray Level Run Emphasis	Second-order
	Busyness	Second-order

## Data Availability

Not applicable.

## Supplementary Materials

The following supporting information can be downloaded at: https://www.mdpi.com/article/10.3390/diagnostics13152573/s1. Table S1: Mann-Whitney significant features in the distinction between responders and non-responders to nCRT for each MRI image at baseline and follow-up.
