# Supplementary material for: Radiomic Features Are Predictive of Response in Rectal Cancer Undergoing Therapy
Source: Diagnostics (Basel). 2023 Aug 2;13(15):2573. doi: 10.3390/diagnostics13152573 (PMC10417449; doi:10.3390/diagnostics13152573)
Supplement: Supplementary file 1 [file diagnostics-13-02573-s001.zip › diagnostics-2492687-supplementary.pdf]

Supplementary Materials

Supplementary Table S1. Mann-Whitney significant features in the distinction between responders and non-responders to nCRT for each MRI image at baseline and follow-up

MRI Phases	Selectedfeatures	Level/Order	p-value
T2 baseline	GrayLevelNonUniformity	Second-order	0.025
	DependenceNonUniformityNormalized	Second-order	0.027
	LargeDependenceEmphasis	Second- order	0.035
	DependenceVariance	Second-order	0.038
	Imc2	Second-order	0.011
	Imc1	Second-order	0.031
	RunVariance	Second-order	0.049
	GrayLevelNonUniformity	Second-order	0.028
	ZoneVariance	Second-order	0.005
	SizeZoneNonUniformityNormalized	Second-order	0.017
	LargeAreaEmphasis	Second-order	0.006
	LargeAreaLowGrayLevelEmphasis	Second-order	0.003
	LargeAreaHighGrayLevelEmphasis	Second-order	0.041
	SmallAreaEmphasis	Second-order	0.022
	Strength	Second-order	0.014
	Busyness	Second-order	0.003
T2 follow-up	LeastAxisLength	Shape	0.010
	Flatness	Shape	0.0011
	DependenceNonUniformity	Second-order	0.045
	SmallDependenceLowGrayLevelEmphasis	Second-order	0.024
	GrayLevelNonUniformity	Second- order	0.045
	RunLengthNonUniformity	Second-order	0.040
	Coarseness	Second-order	0.034
ADC baseline	LeastAxisLength	Shape	0.049
	GrayLevelVariance	Second-order	0.028
	SmallDependenceEmphasis	Second-order	0.009
	SmallDependenceHighGrayLevelEmphasis	Second-order	0.048
	DependenceNonUniformityNormalized	Second-order	0.027
	Idmn	Second-order	0.048
	Contrast	Second-order	0.001
	InverseVariance	Second-order	0.003
	DifferenceVariance	Second-order	0.001
	Idn	Second-order	0.037
	Idm	Second-order	0.005
	Correlation	Second-order	0.023
	SumSquares	Second-order	0.025
	DifferenceAverage	Second-order	0.002
	Id	Second-order	0.002
	GrayLevelVariance	Second-order	0.028
	RunVariance	Second-order	0.037
	LongRunEmphasis	Second-order	0.049
	ShortRunEmphasis	Second-order	0.021
	RunPercentage	Second-order	0.015
	RunLengthNonUniformityNormalized	Second-order	0.036
	SizeZoneNonUniformityNormalized	Second-order	0.008
	ZonePercentage	Second-order	0.018
	SmallAreaEmphasis	Second-order	0.006
	Complexity	Second-order	0.048
	Contrast	Second-order	0.007
ADC follow up	VoxelVolume	Shape	0.042
	MeshVolume	Shape	0.020
	Sphericity	Shape	0.0004
	LeastAxisLength	Shape	0.006
	Elongation	Shape	0.001

	SurfaceVolumeRatio	Shape	0.004
	Flatness	Shape	0.001
	MinorAxisLength	Shape	0.008
	DependenceNonUniformity	Second-order	0.019
	LargeDependenceLowGrayLevelEmphasis	Second-order	0.044
	SmallDependenceLowGrayLevelEmphasis	Second-order	0.015
	LowGrayLevelEmphasis	Second-order	0.010
	ClusterShade	Second-order	0.037
	MaximumProbability	Second-order	0.041
	JointEnergy	Second-order	0.048
	DifferenceEntropy	Second-order	0.012
	SumEntropy	Second-order	0.039
	Imc2	Second-order	0.026
	Uniformity	Second-order	0.032
	Entropy	Second-order	0.015
	ShortRunLowGrayLevelEmphasis	Second-order	0.010
	LowGrayLevelRunEmphasis	Second-order	0.009
	GrayLevelNonUniformityNormalized	Second-order	0.022
	LongRunLowGrayLevelEmphasis	Second-order	0.023
	RunEntropy	Second-order	0.016
	GrayLevelNonUniformityNormalized	Second-order	0.035
	SizeZoneNonUniformity	Second-order	0.022
	SmallAreaHighGrayLevelEmphasis	Second-order	0.048
	LargeAreaLowGrayLevelEmphasis	Second-order	0.030
	LowGrayLevelZoneEmphasis	Second-order	0.008
	SmallAreaLowGrayLevelEmphasis	Second-order	0.032
	Busyness	Second-order	0.036
